# Acne therapy with isotretinoin is well tolerated in patients with established inflammatory bowel disease: a retrospective, single center study

**DOI:** 10.1093/crocol/otag110

**Published:** 2026-10-05

**Authors:** Marley Cutrona, Ahuva Cices, Marla C Dubinsky, Elizabeth A Spencer

**Affiliations:** Department of Dermatology, Icahn School of Medicine at Mount Sinai, New York, NY, United States; Department of Dermatology, Icahn School of Medicine at Mount Sinai, New York, NY, United States; Susan & Leonard Feinstein Inflammatory Bowel Disease Center, Icahn School of Medicine at Mount Sinai, New York, NY, United States; Susan & Leonard Feinstein Inflammatory Bowel Disease Center, Icahn School of Medicine at Mount Sinai, New York, NY, United States; Department of Pediatrics, Division of Pediatric Gastroenterology & Nutrition, Icahn School of Medicine at Mount Sinai, New York, NY, United States; Susan & Leonard Feinstein Inflammatory Bowel Disease Center, Icahn School of Medicine at Mount Sinai, New York, NY, United States; Department of Pediatrics, Division of Pediatric Gastroenterology & Nutrition, Icahn School of Medicine at Mount Sinai, New York, NY, United States

**Keywords:** inflammatory bowel disease, isotretinoin, acne, disease activity, drug safety

## Abstract

**Background:**

Early case reports and observational studies have suggested an association between isotretinoin and inflammatory bowel disease (IBD). However, large cohort studies and meta-analyses have not demonstrated a meaningful association between isotretinoin use and IBD development. Most prior research has focused on incident IBD risk, with limited attention to disease activity among patients with established IBD.

**Methods:**

We conducted a single-center retrospective cohort study of patients with Crohn’s disease (CD) or ulcerative colitis (UC) treated with isotretinoin between 2015 and 2025. Inclusion required at least 1 IBD assessment within the 4 months before and 12 months after isotretinoin initiation. Disease activity was assessed using Harvey–Bradshaw Index (HBI), partial Mayo score (pMS), inflammatory biomarkers, and in some patients, intestinal ultrasound and fecal calprotectin. The primary outcome was composite IBD status change, defined as new clinical activity (Δ ≥ 1 in HBI/pMS), or isotretinoin discontinuation attributed to IBD activity.

**Results:**

Thirty-seven patients met inclusion criteria (57% CD, 43% UC). Most patients were in clinical remission at baseline (86%). Following isotretinoin exposure, 92% remained in remission, with no significant changes in HBI, pMS, erythrocyte sedimentation rate, C-reactive protein, or fecal calprotectin. IBD status change occurred in 4 patients (11%), with events largely attributable to underlying IBD-related factors, including biologic transitions, anti-drug antibody development, or medication dose adjustments.

**Conclusions:**

Among patients with established, well-controlled IBD, isotretinoin was not associated with clinically meaningful worsening of disease activity, supporting its use in carefully selected patients with stable disease.

## Introduction

The relationship between oral isotretinoin and inflammatory bowel disease (IBD) has long been a subject of clinical interest and controversy. Early case reports and selected observational studies raised concern for a potential association between isotretinoin exposure and IBD.^1–3^ However, subsequent large population-based cohort studies, case-control analyses, and meta-analyses have not demonstrated a meaningful association between isotretinoin use and IBD development.^1^^,^^4^ Notably, most of this literature has focused on incident IBD risk, with limited attention to disease activity among patients with established IBD.^5^ In recent acne management guidelines, the American Academy of Dermatology noted that observational studies have not supported an association between isotretinoin and IBD.^6^^,^^7^

Despite this evolving understanding, isotretinoin remains underutilized in patients with IBD.^8^ As a retinoic acid derivative, isotretinoin is the only acne therapy that reliably induces durable remission in moderate-to-severe or refractory nodulocystic acne.^7^ Known adverse effects include mucocutaneous dryness, dyslipidemia, reversible transaminase elevations, teratogenicity, arthralgias, myalgias, rarely rhabdomyolysis, and possible neuropsychiatric effects.^1^^,^^6^^,^^7^ Persistent uncertainty regarding gastrointestinal safety has contributed to therapeutic hesitancy among dermatology and gastroenterology clinicians, often resulting in undertreatment with significant consequences, including reduced quality of life, psychosocial burden, and permanent scarring.^6^^,^^7^ This is increasingly clinically relevant given the expanding use of Janus kinase inhibitors (JAKi) in IBD, which are associated with higher rates of acne, underscoring the need for evidence-based acne management strategies in this population.^8–10^

To address this gap, we present the largest cohort to date of patients with preexisting IBD treated with isotretinoin, evaluating whether isotretinoin therapy is associated with changes in IBD activity.

## Methods

We conducted a single-center retrospective cohort study of patients with IBD at a tertiary-care center who received isotretinoin between 2015 and 2025. The date of isotretinoin initiation was defined as the index date. Inclusion required at least 1 IBD assessment within the 4 months prior to index (baseline) and 1 or more follow-up assessment within 12 months after index. Patients were excluded if isotretinoin start or stop dates were missing from the medical record.

Data on IBD phenotype and concurrent IBD therapy at index were collected. Acne characteristics at index—including prior therapies, anatomic distribution, lesion type, and presence of scarring—were recorded from dermatology documentation where available. IBD activity was assessed using the Harvey–Bradshaw Index (HBI) for Crohn’s disease (CD) and the partial Mayo score (pMS) for ulcerative colitis (UC) for each patient. Objective measures of disease activity obtained as part of routine clinical care were also collected, including biomarkers (erythrocyte sedimentation rate [ESR, mm/hr], C-reactive protein [CRP, mg/L], and fecal calprotectin [FC, μg/g]) and intestinal ultrasound (IUS). IUS was performed by an IBUS-certified gastroenterologist using a standardized protocol; assessments included bowel wall thickness and hyperemia by color Doppler. IUS activity was defined as bowel wall thickness >3 mm and/or hyperemia (modified Limberg score ≥1), assessed at baseline (within 1 month pre-isotretinoin) and again at peak isotretinoin dose. We additionally collected data on temporary or permanent discontinuation of isotretinoin, including whether discontinuation was due to gastrointestinal symptoms or concern for IBD activity as determined by the treating gastroenterologist. All assessments were abstracted from the index date through 3 months following isotretinoin cessation.

The primary outcome was a composite change in IBD status, defined as development of new clinical disease activity (Δ ≥ 1 HBI or pMS) or temporary or permanent discontinuation of isotretinoin attributed to IBD activity by the treating gastroenterologist. Secondary outcomes included clinical remission following isotretinoin exposure (HBI <5 or pMS <2) and changes in clinical indices and inflammatory biomarkers. In a subset of patients with enhanced IBD monitoring while on isotretinoin, including IUS and FC measured at baseline and again at the time of routine dermatology peak-dose laboratory monitoring, within-patient comparisons were used to assess changes in objective IBD activity over time.

Analyses were performed using descriptive summary statistics. Non-parametric tests were used for paired and unpaired comparisons, as appropriate. Time-to-event analyses were evaluated using Kaplan–Meier estimates, with comparisons stratified by IBD subtype where relevant.

## Results

A total of 37 patients met the inclusion criteria. The median age at isotretinoin initiation was 23 years [interquartile range, IQR: 19-28], and the median age at IBD diagnosis was 14 years [IQR: 11-18]. The cohort was predominantly female (65%), and most patients were nonsmokers (76%). Twenty-one (57%) patients had CD, and 16 (43%) had UC. Among patients with CD, a majority (57%) had an uncomplicated phenotype with 52% with ileal involvement. Among patients with UC, half (50%) had extensive or pancolitis. At baseline, tumor necrosis factor (TNF) inhibitor therapy was most common (38%), followed by IL-23 pathway blockade (19%) (Table 1, panel A).

**Table 1:** Demographic and clinical characteristics of patients with inflammatory bowel disease treated with isotretinoin (Panel A, n = 37), and acne characteristics and prior acne treatment among patients with available dermatologic documentation (Panel B, n = 17).

Panel A Demographics and clinical characteristics (*n* = 37)	
**Demographics**	
**Female gender, *n* (%)**	24 (65)
**Smoker, *n* (%)**	9 (24)
**Age (years) at isotretinoin start, median [IQR]**	23 [19-28]
**Age of diagnosis (years), median [IQR]**	14 [11-18]
**Disease duration (years), median [IQR]**	7.6 [5.0-10.3]
**IBD phenotype**	
***Montreal A, n* (%)**	
**1: Age of onset ≤16 years**	23 (62)
**2: Age of onset 17–40 years**	14 (38)
**3: Age of onset >40 years**	0 (0)
**Crohn’s disease**	21 (57)
***Montreal L, n* (%)**	
**1: Terminal ileum**	11 (52)
**2: Colonic**	4 (19)
**3: Ileocolonic**	6 (29)
***Montreal B, n* (%)**	
**1: Nonstricturing, nonpenetrating**	12 (57)
**2: Stricturing**	5 (23)
**3: Penetrating**	4 (19)
***Perianal***	3 (14)
**Ulcerative colitis**	16 (43)
***Montreal E, n* (%)**	
**1: Proctitis**	5 (31)
**2: Left-sided**	3 (19)
**3: Extensive colitis to hepatic flexure**	3 (19)
**4: Pancolitis**	5 (31)
**IBD therapy at baseline, *n* (%)**	
**Anti-TNF (Infliximab, Adalimumab, Golimumab)**	14 (38)
**IL-23 inhibitors (Ustekinumab, Risankizumab)**	7 (19)
**Integrin antagonists (Vedolizumab)**	2 (5)
**JAK inhibitors (Tofacitinib, Upadacitinib)**	3 (8)
**Immunomodulators (6MP, Azathioprine, Methotrexate)**	3 (8)
**5-ASA**	5 (14)
**Budesonide**	1 (3)
**None**	2 (5)

Panel B Acne characteristics and treatment (*n* = 17)	
**Acne distribution, *n* (%)**	
**Face**	16 (94)
**Back**	9 (53)
**Chest**	7 (41)
**Acne lesion type, *n* (%)**	
**Nodulocystic**	6 (35)
**Papulopustular**	2 (12)
**Comedonal**	0 (0)
**Nodulocystic and papulopustular**	2 (12)
**Nodulocystic and comedonal**	1 (6)
**Comedonal and papulopustular**	6 (35)
**Acne features, *n* (%)**	
**Presence of acne scarring**	7 (41)
**IBD medication-induced acne**	3 (18)
**Prior isotretinoin course**	3 (18)
**Acne treatment prior to isotretinoin, *n* (%)**	
**Oral antibiotics (eg, doxycycline, minocycline)**	10 (59)
**Topical antibiotics (eg, clindamycin, benzoyl peroxide)**	8 (47)
**Anti-androgens (eg, spironolactone, clascoterone)**	2 (12)
**Retinoids (eg, tretinoin, adapalene, and tazarotene)**	15 (88)
**Medicated washes (eg, sulfa, benzoyl peroxide)**	4 (24)

Abbreviations: 5-ASA, 5-aminosalicylic acid; IBD, inflammatory bowel disease; IQR, interquartile range; JAK, Janus kinase; TNF, tumor necrosis factor.

The median duration of isotretinoin therapy was 7 months [IQR: 6-10 months]. Overall, 10 patients (27%) received isotretinoin for less than 6 months, while 27 (73%) received treatment for 6 months or longer. Isotretinoin dosing was inconsistently documented; however, among patients with available dosing data (*n* = 32), 28 (88%) received a daily dose greater than 30 mg.

### Acne characteristics of the cohort

Among the 46% (17/37) of patients with available acne phenotypic data, 77% (13/17) had received ≥2 acne therapies prior to initiating isotretinoin. The most common prior treatments were topical retinoids (15/17, 88%) and doxycycline (10/17, 59%); 18% (3/17) of patients reported acne that was induced or exacerbated by IBD-directed therapies, including JAKi or corticosteroids. Almost all patients had facial acne (16/17, 94%), and 41% (7/17) had evidence of acne scarring (Table 1, panel B).

### IBD status change following isotretinoin

Most patients had well-controlled IBD prior to isotretinoin initiation, with 86% (32/37) in clinical remission. Following isotretinoin exposure, 34/37 patients (92%) were in clinical remission, with no significant changes in HBI, pMS, ESR, CRP, or FC (Table 2).

**Table 2:** Isotretinoin outcomes.

Isotretinoin outcomes (*n* = 37)	
**Composite IBD clinical status change after isotretinoin**	4 (11)
**Median isotretinoin duration, weeks**	31 (26-46)

Clinical parameters	Baseline	Postisotretinoin	*P*-value^a^
**Clinical remission (*n* = 37)**	32 (86)	34 (92)	0.7
**HBI (*n* = 21)**	0 [0-0]	0 [0-0]	0.3
**Partial Mayo score (*n* = 16)**	0 [0-1.3]	0 [0-0.3]	0.8
**Erythrocyte sedimentation rate (*n* = 32)**	11 [7-19]	8 [5-21]	0.2
**C-reactive protein (*n* = 36)**	2.2 [0.8-3.0]	2.0 [0.9-3.0]	1.0
**Calprotectin (*n* = 9)**	147 [64-228]	158 [35-275]	0.3

Medians [IQR] presented for continuous variables; *n* (%) for categorical.

Abbreviations: HBI, Harvey–Bradshaw Index; IBD, inflammatory bowel disease; IQR, interquartile range.

a Paired continuous variables were analyzed using Wilcoxon signed-rank tests, and paired categorical outcomes were compared using McNemar’s test.

The composite primary outcome of IBD status change—defined as the development of clinical disease activity (Δ ≥ 1 HBI/pMS) or temporary or permanent isotretinoin cessation initially attributed to IBD activity by the treating gastroenterologist—occurred in 4/37 patients (11%) (2 CD and 2 UC), with no difference in event-free survival by IBD type (log-rank *P* = .85).

Among these 4, 2 completed isotretinoin, 1 temporarily discontinued therapy, and 1 permanently discontinued therapy. In patients who completed isotretinoin, status changes were attributable to biologic transitions or anti-drug antibody–mediated loss of response. One patient briefly paused isotretinoin after a single episode of rectal bleeding, attributed to hemorrhoidal disease or mucosal dryness, and resumed isotretinoin without recurrence. Another patient developed worsening IBD symptoms during isotretinoin initiation concurrent with upadacitinib dose reduction (45-30 mg); isotretinoin was discontinued, symptoms resolved after upadacitinib dose escalation, and isotretinoin was not rechallenged.

### Objective monitoring before and after isotretinoin

A cohort of 7 patients (CD = 5, UC = 2) had both IUS and FC before isotretinoin initiation and at peak dose. In those, 71% (5/7) were in IUS remission before and after isotretinoin; the remaining 29% (2/7) had IUS activity at baseline that resolved while on isotretinoin. The median change in FC was 99 µg/g (IQR: −27-133), which did not reach statistical significance (Wilcoxon signed-rank test, *P* = .078).

## Discussion

In this cohort of patients with established, predominantly well-controlled IBD treated with isotretinoin, we observed no signal that isotretinoin exposure was associated with clinically meaningful worsening of IBD activity. Clinical indices, inflammatory biomarkers, and available objective measures remained stable, and the small number of observed IBD status changes were attributable to underlying IBD-related factors—including biologic transitions, anti-drug antibody development, or medication dose adjustments—rather than isotretinoin exposure. By directly evaluating clinical, biochemical, and imaging outcomes in patients with known IBD on isotretinoin, our findings address a gap in prior literature that has largely focused on incident IBD risk,^1–3^ and provide disease-activity-focused reassurance regarding isotretinoin use in appropriately selected patients with well-controlled IBD.

Our findings are consistent with a prior report by Lopez et al. evaluating isotretinoin exposure in patients with IBD, which found no statistically significant association between isotretinoin use and IBD flares. That work suggested that patients with recent disease instability may be at higher risk of subsequent activity, highlighting baseline disease control as a key determinant of outcomes with isotretinoin in patients with IBD.^5^ Taken together, these data suggest that underlying IBD-related factors play a dominant role in determining IBD activity and that observed changes during isotretinoin therapy should be carefully contextualized before attributing causality to isotretinoin exposure.

These findings should be interpreted within the context of several limitations. This study is limited by its retrospective design, modest sample size, and the predominance of patients with quiescent disease at isotretinoin initiation. Paired objective monitoring with fecal calprotectin and IUS was available in only a small subset of patients. Although CRP was available for most patients, it can remain normal in approximately one-third of patients with CD, particularly those with ileal disease.^11^ Colonoscopy and magnetic resonance enterography were not routinely performed at time points corresponding to isotretinoin initiation and cessation. Thus, objective disease stability could not be confirmed across the entire cohort. Prospective, multicenter studies incorporating objective measures of disease severity and including patients with active disease are needed, as these findings are most generalizable to patients with well-controlled IBD.

Despite these limitations, the clinical implications of our findings are particularly relevant in the current treatment landscape, where acne is increasingly encountered as an adverse effect of IBD-directed therapies, particularly JAKi and corticosteroids. Although recent gastroenterology-focused guidance has appropriately emphasized recognition and management of drug-induced acne, isotretinoin decisions were recommended to be deferred to dermatology.^8^ In practice, however, initiation often hinges on explicit gastroenterology “clearance,” placing responsibility for risk assessment on the IBD provider.

In this real-world context, uncertainty or excessive caution by gastroenterologists may delay or prevent access to effective acne therapy, with meaningful consequences for patient quality of life and treatment adherence.^10^^,^^12^ Our findings directly inform this decision-making process by providing disease-activity-specific data in patients with established IBD, enabling gastroenterologists to confidently counsel patients and collaborating dermatologists regarding isotretinoin safety.

## Conclusion

Isotretinoin was not associated with clinically meaningful worsening of disease activity in this cohort of patients with predominantly well-controlled IBD. Variations in IBD course during treatment were likely related to underlying IBD-related factors, underscoring the importance of cautious interpretation before assigning causality to isotretinoin. These findings suggest that isotretinoin may be considered in carefully selected patients with stable IBD through multidisciplinary collaboration between dermatologists and gastroenterologists.

## Data Availability

Data are available on reasonable request to the study authors.

## References

[1] Yu CL , Chou PY, Liang CS, et al Isotretinoin exposure and risk of inflammatory bowel disease: a systematic review with meta-analysis and trial sequential analysis. Am J Clin Dermatol. 2023;24:721-730. 10.1007/s40257-023-00765-9 37010780

[2] Passier JL , Srivastava N, van Puijenbroek EP. Isotretinoin-induced inflammatory bowel disease. Neth J Med. 2006;64:52-54. 16517990

[3] Reddy D , Siegel CA, Sands BE, Kane S. Possible association between isotretinoin and inflammatory bowel disease. Am J Gastroenterol. 2006;101:1569-1573. 10.1111/j.1572-0241.2006.00632.x 16863562

[4] Etminan M , Bird ST, Delaney JA, Bressler B, Brophy JM. Isotretinoin and risk for inflammatory bowel disease: a nested case-control study and meta-analysis of published and unpublished data. JAMA Dermatol. 2013;149:216-220. 10.1001/jamadermatol.2013.1344 23426479

[5] Lopez CG , Kwak R, McCormack L, Zhou G, Charrow A. Impact of isotretinoin on inflammatory bowel disease flare in patients with preexisting inflammatory bowel disease diagnosis: a cross-sectional study. J Am Acad Dermatol. 2022;87:1399-1400. 10.1016/j.jaad.2022.05.024 35597387

[6] Association AAoD. Position statement on isotretinoin. Approved December 9, 2000; Amended March 25, 2003; March 11, 2004; November 13, 2010; February 19, 2018; Revised May 3, 2025.

[7] Reynolds RV , Yeung H, Cheng CE, et al Guidelines of care for the management of acne vulgaris. J Am Acad Dermatol. 2024;90:1006.e1001-1006.e1030. 10.1016/j.jaad.2023.12.017 38300170

[8] Temido MJ , Honap S, Bursztejn AC, et al Drug-induced acne in inflammatory bowel disease: a practical guide for the gastroenterologist. Am J Gastroenterol. 2025;120:125-134. 10.14309/ajg.0000000000003119 39382676

[9] Martinez J , Manjaly C, Manjaly P, et al Janus kinase inhibitors and adverse events of acne: a systematic review and meta-analysis. JAMA Dermatol. 2023;159:1339-1345. 10.1001/jamadermatol.2023.3830 PMC10585588 37851459

[10] Spencer EA , Smyth M, Cices A, et al Prevalence, age-distribution, and impact on management of acne in patients on upadacitinib for inflammatory bowel disease: insights from a single-center survey. Crohns Colitis 360. 2026;8:otag036. 10.1093/crocol/otag036 PMC13161555 42131777

[11] Torres J , Mehandru S, Colombel J-F, Peyrin-Biroulet L. Crohn’s disease. Lancet. 2017;389:1741-1755. 10.1016/S0140-6736(16)31711-1 27914655

[12] Spencer EA , Smyth M, Cices A, et al Prevalence, age-distribution, and impact on management of acne in patients on upadacitinib for inflammatory bowel disease: insights from a single-center survey. Crohn’s Colitis. 2025;8:otag036. 10.1093/crocol/otag036 PMC13161555 42131777

